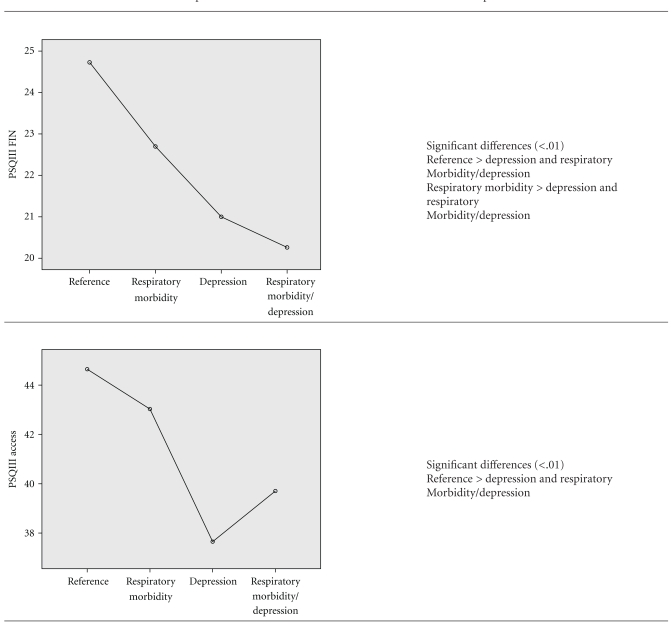# Determining Satisfaction with Access and Financial Aspects of Care for Persons Exposed to Libby Amphibole Asbestos: Rural and National Environmental Policy Implications

**DOI:** 10.1155/2011/789514

**Published:** 2011-10-04

**Authors:** Charlene A. Winters, Wade Hill, Sandra W. Kuntz, Clarann Weinert, Kimberly Rowse, Tanis Hernandez, Brad Black

**Affiliations:** ^1^College of Nursing, Montana State University, Missoula Campus, 32 Campus Drive 7416, Missoula, MT 59812-7416, USA; ^2^College of Nursing, Montana State University, Sherrick Hall, Bozeman Campus, P.O. Box 173560, Bozeman, MT 59717-3560, USA; ^3^Center for Asbestos Related Disease, 214 E. 3rd Street Libby, MT 59923, USA

## Abstract

Libby, Montana is a Superfund site and epicenter of one of the worst environmental disasters in the USA history in terms of asbestos-related mortality and morbidity. Perceptions of access and financial aspects of care were explored among a national cohort of persons postasbestos exposure and prior to a 2009 Public Health Emergency Declaration. Our findings indicated the Libby cohort was significantly less satisfied with access and financial aspects of care as measured by two PSQ-III scales when compared to an adult, chronically ill patient sample. Participants with higher levels of respiratory morbidity and depression had significantly lower satisfaction scores.

## 1. Introduction

The rural community of Libby, Montana is the epidemiological epicenter of asbestos-related disease (ARD) with mortality rates 40–80 times higher when compared to rates in Montana and the USA [1]. What began as a study of excess mortality related to occupational exposure at a mining site soon included respiratory disease in household members and residents with only transient or ambient community contact to asbestos [2]. In 2008, Libby was described as the worst environmental disaster in US history [3]. The state of federal involvement in the technological and slow-motion events that exposed community residents and occupational workers to Libby amphibole asbestos [4], included public health assessments [5], enhanced regulation [6, 7], protective policy recommendations [8, 9], and a 2009 public health emergency declaration—the first and only Superfund site in the country given this designation [10]. This action by the US Environmental Protection Agency (EPA) represented a major step in addressing the aftermath of amphibole asbestos exposure in a community first placed on the National Priorities List as a Superfund site in 2002 [11]. The EPA established the emergency declaration under the Comprehensive Environmental Response, Compensation, and Liability Act (CERCLA) and in so doing recognized the public health impact and need for additional environmental cleanup and health care access support for residents “who have been exposed or may be exposed” to Libby amphibole asbestos (¶ 2) [12].

Two years prior to the June 17, 2009 Public Health Emergency Declaration, the Libby Health Status Study (LHSS) was conducted to establish a more comprehensive understanding of the biopsychosocial health status and health service needs for persons exposed to Libby amphibole asbestos. One of the principal aims of the study was to evaluate access, availability, convenience, and financial aspects of care among the national Libby cohort [13]. At the time of this study, access to care was described as less than comprehensive with a patchwork of services distributed unevenly to a population with both detectable disease and those exposed but still undiagnosed due to the 10-plus year latency period of ARD. The purpose of this paper is to (a) report satisfaction with access, availability, convenience, and financial aspects of care; (b) explore differences in satisfaction with access/financial aspects of care and increasing respiratory morbidity and depression; (c) identify rural health policy implications for a national cohort of persons exposed to Libby amphibole asbestos.

**Figure 1:**
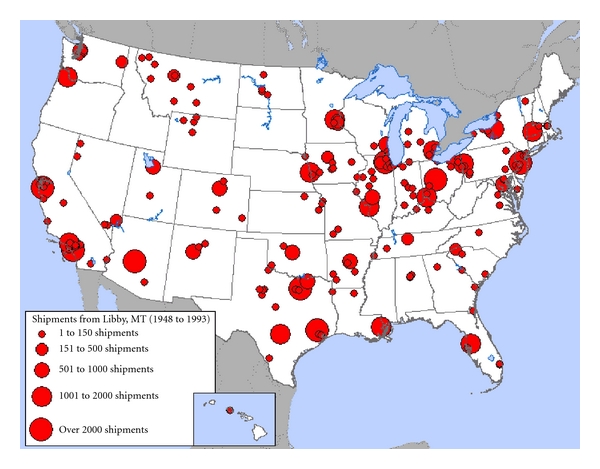
US Shipments from Libby, MT (1948–1993). Copyright © Environmental Working Group, http://www.ewg.org. Reprinted with permission.

Exposure to Libby amphibole asbestos can be traced to mining, processing, and distributing contaminated vermiculite ore from Libby, Montana to over 250 sites across the USA (Figure 1) and sites around the world. During the years the mine was in operation (1919–1990), millions of tons of ore were produced providing nearly 80% of the world's supply of vermiculite. It is estimated that the raw ore contained as much as 26% naturally occurring amphibole asbestos [5]. Vermiculite is flat and shiny in its natural state and puffed and dull when expanded by heat. The expanded or “popped” form made the material suitable for use in many commercial applications including building insulation, fire-proofing, and as a soil amendment [14]. Attic insulation contaminated with Libby amphibole asbestos may still be in place in schools, businesses, and as many as 35 million homes around the USA alone [6, 15].

In addition to the occupational exposure associated with vermiculite mining and handling, workers, household members, and residents of Libby were exposed when the product was distributed throughout town and used for gardening, insulation in homes and schools, additives for driveways, and a school baseball field [16]. Air sampling in Libby in the 1980s identified amphibole asbestos fibers in excess of occupational limits of 0.1 fiber/cm^3^ over eight hours set by the US Occupational Safety and Health Administration (OSHA) [17, 18]. As early as 1986, a cohort study (1963–1983) detected increased mortality rates among Libby vermiculite mine workers [19]. The mine closed in 1990 but it would be another nine years before the community-wide exposure was addressed by federal authorities. In June of 2008 [20], updated asbestosis mortality statistics were released for 1995–2004. Lincoln County Montana, where Libby is located, had the highest age-adjusted asbestosis death rate per million in the USA for residents ages 15 and older.

Environmental protocols were put into place to begin the process of decontaminating homes, schools, and public sites within the Libby community. Medical screenings sponsored by the US Agency for Toxic Substances and Disease Registry (ATSDR) were conducted in 2000 and 2001 to assess the presence of lung abnormalities that may be related to asbestos exposure [21]. Those eligible for screening (interviews, chest X-ray, and respiratory spirometry testing) included former mine workers from Libby and persons who lived, worked, or played in Libby for at least six months prior to December 31, 1990. A total of 7,307 participated in the two rounds of testing. In 2000, a health center was established in Libby devoted to the diagnosis, treatment, and monitoring of persons exposed to Libby amphibole asbestos [22]. From 2003 to 2008, the Montana Department of Public Health and Human Services (MT DPHHS) conducted a screening program with ATSDR funding and technical support [21]. Also beginning in 2003, medical screening was available to residents who met eligibility criteria for the Libby Asbestos Medical Plan (LAMP) [23], a program funded by a legal settlement between the US Environmental Protection Agency and the vermiculite mine owner, and the State of Montana. LAMP served as an insurance of last resort in the absence of federal- or state-supported insurance. LAMP provided support for health screening and services not provided under the voluntary medical program provided by the mining company [24]. Individuals qualified for the voluntary medical program that lived and worked within a 20-mile radius of the Libby mine or mill site for at least 12 consecutive months prior to January 1, 2000 and had a qualifying medical condition. Under the voluntary health plan managed by Health Network of America (HNA), coverage for basic services such as oxygen and other medical treatments could be denied when reviewers determined that treatment was not directly related to amphibole asbestos exposure. These denials were made despite the attending physician's diagnosis and treatment plan. This patchwork of medical services resulted in care that was episodic and fragmented (personal communication, Libby health care worker, September 4, 2008).

## 2. Materials and Methods

### 2.1. Study Framework

 Guidance related to the study aims was available from three sources. First, the literature on *adaptation to chronic illness *[25–29] and previous work of team members informed this study [30–35]. Adaptation to a chronic illness is a complex process involving internal and external factors [26]. Taken as a whole, these influences affect a person's ability to successfully adapt to a chronic illness like ARD. The key components of adaptation are (a) environmental stimuli, (b) psychosocial response, and (c) illness management. This study focused on the psychosocial response to illness and the perceived availability of health care services.

Second, the *Care Across the Continuum Framework *[36] developed by the Rural Policy Research Institute at the University of Nebraska supported examination of modalities for delivering services to rural populations. Applying recommendations from the US Institute of Medicine (IOM) Committee on Quality of Health Care in America [37] to the rural population, the following tenets of primary care were acknowledged: (a) care is comprehensive—any problem at any stage in life is addressed; (b) care is coordinated—a combination of health services and information is provided and ordered rationally; (c) care is continuous—a team of providers deliver care across time; (d) care is accessible—patients can initiate an interaction for any health problem, overcoming any barriers such as geography, financing, and culture [38].

The third source of guidance came from *Healthy People 2010 *and *Rural Healthy People 2010: A Companion Document to Healthy People 2010 *[39]. The ARD population is primarily male and between the ages of 55 and 84 years [40]. In the absence of a comprehensive understanding of the biopsychosocial health status and the continuum of care/disease progression that tracks gaps in services, objectives related to access to services for rural populations, and primary care for aging populations are particularly cogent. 

Additionally, recent literature pointed to the need for access to care oversight when communities and residents have experienced a disaster. Shehab et al. [41] highlighted the need for policies addressing mental and physical health issues when individuals are displaced due to a natural disaster. The authors found high levels of depression and physical disorders in individuals relocated to US Federal Emergency Management Agency (FEMA) travel trailer parks during and in the long aftermath of Hurricane Katrina [41]. Another study found specialty care out of reach for uninsured or underinsured individuals even with the safety net provided by teaching hospitals where initial access was assured [42]. Libby residents with ARD often experienced the opposite outcome. At the time of the study, specialty care was available to residents for asbestos-related illnesses through the Center for Asbestos Related Disease (CARD), but insurance coverage was not available through the mining company supported health plan (HNA) for primary care of comorbidities such as congestive heart failure, autoimmune disorders, or asthma considered unrelated to asbestos exposure (Personal communication, Libby health care provider, September 4, 2008).

### 2.2. Study Design

A descriptive cross-sectional study design was used by the research team to explore the biopsychosocial health status and perceived satisfaction with access/financial aspects of care among local and distant patients of the CARD clinic located in Libby, Montana. CARD provides long-term asbestos health screening, monitoring, ARD diagnosis, specialized asbestos healthcare, and counseling to local and distant people affected by Libby amphibole asbestos. Two of the seven subscales of the Medical Outcomes Study, Patient Satisfaction Questionnaire (PSQ-III) were used to determine perception of and satisfaction with access to care and financial aspects of care. First, the 12-item Access, Availability, and Convenience subscale was used to measure perception of availability of medical resources, waiting times, and continuity of care (Cronbach's alpha (*α*) 0.86). Second, Financial Aspects of Care was measured with the 8-item subscale that assessed perception of difficulty in paying for medical care (*α* = 0.89) [43–46]. In addition to the PSQ-III survey results reported here, two other measures were included in the participant questionnaire. The St. George's Respiratory Questionnaire (SGRQ) (*α* = .90–.95) was used to assess respiratory health-related quality of life and impact of illness in individuals with lung disease [47]. The Center for Epidemiological Studies Depression Scale (CES-D) was used to measure depression (*α* = .84–.90) [48] (see Table 1 for details). Finally, to reduce participant burden, demographic data (age, gender, marital status, residence, and insurance coverage), degree of respiratory illness, and route of exposure were collected from CARD patient records.

### 2.3. Participants

The total number of individuals exposed to Libby amphibole asbestos is unknown due to the widespread distribution of the contaminated vermiculite ore. For the study reported here, a cohort of 426 persons from a total of 1500 CARD patients participated in the study. Of these, most were *local *residents from the Libby area, (*n* = 286; 67.1%) and a smaller number were *distant *patients living in a community elsewhere in the USA (*n* = 140; 32.9%). All participants had a history of exposure to Libby amphibole asbestos; most were married (*n* = 310, 72.8%) men (*n* = 242, 56.8%), with an age range from 20 to 49 (*n* = 52, 12.2%); 50 to 64 (*n* = 207, 48.6%); or 65+ (*n* = 167, 39.2%). Insurance information was collected for each participant with nearly one-quarter (*n* = 100; 23.5%) reporting some type of public program (Medicare, Medicaid, Veterans Administration, or Social Security Disability) or private insurance (*n* = 114; 26.8%); approximately half of all participants (*n* = 212; 49.8%) reporting coverage through the HNA (voluntary) mine plan or LAMP. (see Table 2 for demographic details).

### 2.4. Procedure

 To publicize the study, a notice was placed in the CARD newsletter and descriptive posters and brochures that stated the study purpose were available in the CARD clinic waiting room. On an individual basis, clients presenting to the clinic were approached and asked to participate in the study. Those who consented completed either a one-time electronic (computer-based) or paper/pencil questionnaire during their clinic visit or took it home to complete with a stamped self addressed return envelope to CARD. In addition, CARD patients who were exposed to Libby amphibole asbestos and lived elsewhere in the USA were invited to participate in the study via mail by including a letter describing the study, consent form, and a paper copy of the questionnaire and return envelope with normal clinic correspondence. Participants completed the study questionnaire independently or with assistance from family members or the research assistant when needed. Clinic staff provided relevant health status and demographic information for all participants. Data were collected from February to September, 2007. 

### 2.5. Procedure

Questionnaire data collected electronically at the CARD clinic were transmitted directly to a protected database at the research office through a secure Internet connection. The paper/pencil questionnaire data and the deidentified health status and demographic information provided by clinic staff were entered into an electronic form by the research assistant and were also sent to the research office via the secure Internet site. All data were identified by a unique study code number and exported into SPSS (Chicago, Ill, USA) on a weekly basis. Deidentified summary results from the CES-D were returned to CARD on a weekly basis. Clinic staff used the unique participant study number to match the summary results to the correct client, and then filed the results in the client's health record for use in the clients' plan of care. 

### 2.6. Protection of Human Subjects

The procedures of this study were approved by the University Institutional Review Board. Written informed consent was obtained from all participants prior to data collection. Each participant was assigned a unique study code number and deidentified data were used in the analysis. The research team members remained blinded to the identity of each participant.

## 3. Results

The overall mean score for satisfaction with access to care was 42.5 (sd = 7.0), and for financial aspect of care 22.8 (sd = 7.2). A one-way ANOVA was used to examine differences in satisfaction with access and financial aspects of healthcare according to residence, gender, source of asbestos exposure, age, and primary insurance status (see Table 3). The ANOVA summary table indicated that omnibus differences in satisfaction with access to care were found only for age *F*(2, 423) = 10.5, *P* < 0.05. Post hoc analyses using the Tukey HSD test indicated that participants in the youngest two age categories (0–49 years and 50–64 years) were significantly less satisfied with access to care than those in the oldest age category of 65+ years. The ANOVA analysis was repeated examining satisfaction with financial aspects of care and omnibus differences were found among three variables including exposure status, *F*(2, 423) = 4.58, *P* < 0.05, age *F*(2, 423) = 37.1, *P* < 0.05, and source of primary insurance, *F*(2, 423) = 11.6, *P* < 0.05. Exposure through a family member or household contact (versus other routes of exposure), younger age, and having a primary source of insurance through the voluntary HNA (mining) plan or LAMP programs resulted in the lowest scores on satisfaction with financial aspects of care.

One-sample *t*-tests were performed to examine the differences between overall means for each subscale in this study for comparison to results from chronically ill patients reported in the literature (Table 4). Findings indicated that the Libby sample scored significantly lower on the access subscale (*t* = −10.63, *df* = 467, *P* = .000) as well as the financial subscale (*t* = −19.04, *df* = 468, *P* = .000) as compared with a sample (*n* = 2, 197) of adult patients (age 18–108) with one or more of four chronic conditions [46].

This study also explored differences in perceived access and financial aspects of care (Tables 5 and 6) related to respiratory morbidity and depression. For this analysis, respondents were categorized into 4 groups including (1) lowest 50th percentile SGRQ and lowest 50th percentile CES-D (reference category), (2) highest 50th percentile SGRQ and lowest 50th percentile CES-D (respiratory morbidity only), (3) lowest 50th percentile SGRQ and highest 50th percentile CES-D (depression only), and (4) highest 50th percentile SGRQ and highest 50th percentile CES-D (both respiratory morbidity and depression). The ANOVA analysis revealed that omnibus differences for satisfaction with both access to care, *F*(3, 431) = 18.9, *P* < 0.05, and financial aspects of care, *F*(3, 431) = 10.6, *P* < 0.05, were present. Those subjects scoring low on depression and respiratory morbidity (i.e., reference category) had significantly higher scores on both satisfaction with access to care and financial aspects of care as compared with those subjects scoring high on depression alone or depression in combination with respiratory morbidity. Additionally, for satisfaction with access to care, those scoring high on respiratory morbidity only were more satisfied than those with both respiratory morbidity and depression.

## 4. Discussion

Victims of one of the worst environmental disasters in US history may be challenged to realize the recommendations of the Institute of Medicine's Committee on Quality of Health Care in America [37] suggesting health care should be comprehensive, coordinated, continuous, and accessible to rural residents. The presence of the CARD clinic providing ARD specialty care services may serve as a stopgap and somewhat of an equalizing factor for the provision of care to both local and distant patients [29]; however, when compared to persons with other chronic illnesses, the Libby cohort was significantly less satisfied with access and financial aspects of care. Among the Libby cohort, younger participants were less satisfied with access and financial aspects of care than older members, while exposure through a family member of household contact (versus other routes of exposure) and having a limited source of insurance through the voluntary HNA (mining) or LAMP programs resulted in the lowest scores on satisfaction with financial aspects of care. It is also important to note that the tool measuring satisfaction with care did not distinguish between specialty and primary health care. 

These findings are understandable given that nearly 50% of participants had limited health insurance available from the voluntary HNA (mining) plan funded by the company who owned the vermiculite mine and younger participants would not be eligible for federal medical insurance (Medicare). Persons experiencing other technological disasters (e.g., radiation leak from a damaged nuclear power plant, dam collapse, and toxic landfill) report victimization, loss of control, uncertainty [49–51], conflict, controversy, social division, stigma [52], and distrust of societal institutions on which we all depend [53]. Conflict and stigma have specifically been found among persons exposed to Libby amphibole asbestos [54]. More research is needed among persons exposed to Libby amphibole asbestos to determine the relationship between emotions and satisfaction with access to care. 

Depression was also found to negatively influence perceived satisfaction with access and financial aspects of care. Depression results in sadness, loss of interest in a person's usual activities, feelings of worthlessness or hopelessness, disturbed sleep or appetite, low energy, and poor concentration [55]. Depression is one of the most widespread health conditions and is expected to be second only to heart disease as the source of global burden of disease by 2020 [56]. More than one in twenty Americans experience depression [57] and it is increasingly viewed as a chronic illness because of its high rate of symptom recurrence [58]. Depression has great potential for disrupting the lives of affected individuals, influencing their personal and family relationships, productivity in employment and personal lives, and ultimately, the community at large. As a social health condition, depression is linked to suicide, alcohol and drug misuse, and a variety of chronic, health impairing behaviors [59]. Likewise, depression can inhibit the effective management of illness and health promotion behaviors. Authors have documented anxiety, chronic stress, and depression among environmental disaster victims exposed to nuclear radiation and toxic chemicals [49–52]. While specific comparisons of depression among the Libby amphibole asbestos cohort and victims of other environmental disasters are not possible; given that different tools were used to measure depression, it is clear that psychological distress is to be anticipated in persons experiencing environmental disasters. 

## 5. Conclusions

Results of this study are significant for those experiencing a slow-motion environmental disaster but could also apply to other technological (e.g., Gulf Coast Oil Spill) or natural disasters (hurricanes, earthquakes, etc.) with long-term sequelae. Policy is a process that creates social change for health. “Public policy is the way a society frames what it wishes to become” [60, page 1]. Several observations with policy implications bear mentioning.

1. Uncertainty about both access and availability of resources necessary to manage long-term and lifelong physical, mental, social, or economic health complicate the effects of a community-wide disaster.
2. Experience caring for or supporting loved ones who may struggle with a lengthy illness, access to care issues, or financial challenges may create additional family burdens, especially when the disaster impacts multiple generations in a single community.
3. Comorbidities like depression could influence perception of health care access and financial ability to cope with a disaster-related event.
4. Lengthy delays in state and federal government interventions can add to insecurities and hopelessness.

Hope for the future in the community of Libby includes the first EPA Public Health Emergency declaration under the CERCLA (June 17, 2009), and new legislation in the Affordable Care Act (2010) represents a significant federal public policy change to address needed amphibole asbestos-related care to Libby residents. This study of perceived access and financial aspects of care conducted prior to the public health emergency declaration serves as a baseline measure for comparison with future, postdeclaration studies of satisfaction with care. Repeating the PSQ-III in the future could serve as a valuable indicator of one federal policy decision.

## Figures and Tables

**Table 1:** Measures.

Concept	Measure	Number of items	Reported alpha	Study alpha
Depression	Center for Epidemiological Studies	20	.84–.90	.89
Access/availability/convenience	Patient Satisfaction Questionnaire—subscale	12	.86	.81
Financial aspects	Patient Satisfaction Questionnaire—subscale	8	.89	.89
Health impairment and quality of life in respiratory disease	St. George Respiratory Questionnaire	76	.90–.95	.89

**Table 2:** Demographics: sample characteristics (*n* = 426).

Participant characteristics	Subjects	Sample %
Marital status		
Married	310	72.8
Single	34	8.0
Widow(er)	38	8.9
Divorced	40	9.4
Separated	4	0.9
Primary insurance		
Public (Medicare, Medicaid, Va, SSI)	100	23.5
Private	114	26.8
HNA/LAMP	212	49.8
Gender		
Men	242	56.8
Women	184	43.2
Age		
20–49	52	12.2
50–64	207	48.6
65+	167	39.2
Location		
Local	286	67.1
Distant	140	32.9

**Table 3:** Satisfaction with access and financial aspects of care (*n* = 426).

	PSQ-III access means (SD)	*F*/*P*	Pair diffs.	PSQ-III financial means (SD)	*F*/*P*	Pair diffs.^1^
Overall	42.5 (7.0)			22.8 (7.2)		
Residence						
Local	42.9 (6.7)	2.7/ns	NA	22.9 (7.2)	0.18/ns	NA
Distant	41.8 (7.6)			22.5 (7.3)		
Gender						
Men	42.6 (7.2)	0.03/ns	NA	23.2 (7.0)	2.00/ns	NA
Women	42.5 (6.8)			22.3 (7.4)		
Exposure						
Worker (1)	40.7 (9.7)	3.5/<.05	NA	22.4 (7.4)	4.58/<.05	2 versus 3
Family/HH Contact (2)	41.7 (6.9)			21.3 (7.1)		
Other (3)	43.2 (6.7)			23.5 (7.1)		
Age						
20–49 (1)	40.3 (6.6)	10.5/<.01	1 versus 3 2 versus 3	18.8 (6.8)	37.1/<.01	1 versus 3 2 versus 3
50–64 (2)	41.7 (7.2)			21.1 (6.8)		
65+ (3)	44.2 (6.7)			25.9 (6.6)		
Primary insurance						
Public (1)	42.8 (6.9)	2.6/ns	NA	23.3 (7.1)	11.6/<.01	1 versus 3 2 versus 3
Private (2)	43.6 (6.6)			25.0 (6.8)		
HNA/LAMP (3)	41.9 (73)			21.3 (7.2)		

^1^Tukey HSD *P* < 0.05.

**Table 4:** Comparison of mean scores for perceived access and financial aspects of care.

PSQ-III subscale	Subscale score range		Comparison mean (SD)	Libby group mean (SD)	*t*	*df*	*P*
	Low–high						
Access	12	60	46.03 (7.23)	42.54 (7.03)	−10.63	467	<.01
Financial	8	40	29.10 (6.90)	22.75 (7.21)	−19.04	468	<.01

**Table 5:** Differences in perceived access and financial aspects of care (*n* = 426).

	*n*	Access means (SD)	Financial means (SD)
Reference (1)	191	44.6 (6.2)	24.7 (6.8)
Resp. morbidity only (2)	99	43.0 (6.5)	22.7 (7.5)
Depression only (3)	26	37.7 (7.2)	21.0 (7.2)
Resp. morbidity + Depression (4)	110	39.7 (7.1)	20.3 (7.0)
*F*		18.9	10.6
*P*		<.01	<.01
Pair diffs.^1^		1 versus 3	1 versus 3
		1 versus 4	
		2 versus 3	1 versus 4
		2 versus 4	

^1^Tukey HSD *P* < 0.05.

**Table 6:** Comparison of means for satisfaction with access and financial aspects of care.